# β-*N*-Methylamino-l-alanine (BMAA) perturbs alanine, aspartate and glutamate metabolism pathways in human neuroblastoma cells as determined by metabolic profiling

**DOI:** 10.1007/s00726-017-2391-8

**Published:** 2017-02-04

**Authors:** Mikael K. R. Engskog, Lisa Ersson, Jakob Haglöf, Torbjörn Arvidsson, Curt Pettersson, Eva Brittebo

**Affiliations:** 1 Division of Analytical Pharmaceutical Chemistry, Uppsala University, Box 574, 751 23 Uppsala, Sweden; 2 Department of Pharmaceutical Biosciences, Uppsala University, Box 591, 751 23 Uppsala, Sweden; 3 Medical Product Agency, Box 26, Dag Hammarskjölds väg 42, 751 03 Uppsala, Sweden

**Keywords:** Neurotoxin, MS, NMR, Global metabolite profiling, Metabolism, BMAA

## Abstract

β-Methylamino-l-alanine (BMAA) is a non-proteinogenic amino acid that induces long-term cognitive deficits, as well as an increased neurodegeneration and intracellular fibril formation in the hippocampus of adult rodents following short-time neonatal exposure and in vervet monkey brain following long-term exposure. It has also been proposed to be involved in the etiology of neurodegenerative disease in humans. The aim of this study was to identify metabolic effects not related to excitotoxicity or oxidative stress in human neuroblastoma SH-SY5Y cells. The effects of BMAA (50, 250, 1000 µM) for 24 h on cells differentiated with retinoic acid were studied. Samples were analyzed using LC–MS and NMR spectroscopy to detect altered intracellular polar metabolites. The analysis performed, followed by multivariate pattern recognition techniques, revealed significant perturbations in protein biosynthesis, amino acid metabolism pathways and citrate cycle. Of specific interest were the BMAA-induced alterations in alanine, aspartate and glutamate metabolism and as well as alterations in various neurotransmitters/neuromodulators such as GABA and taurine. The results indicate that BMAA can interfere with metabolic pathways involved in neurotransmission in human neuroblastoma cells.

## Introduction

β-Methylamino-l-alanine (BMAA) is a neurotoxic non-proteinogenic amino acid produced by ubiquitous organisms such as cyanobacteria, diatoms and dinoflagellates in terrestrial, marine, brackish and fresh water environments (Jiang et al. 2014; O’Neil et al. 2012; Paerl and Paul 2012). It has potential to biomagnify in a terrestrial food chain, and to bioaccumulate in fish and shellfish (Jiang et al. 2014). This amino acid is considered as a potential health risk because of its putative role in neurodegenerative diseases (Banack and Cox 2003). Recent studies have demonstrated that long-term oral exposure of vervet monkeys to BMAA triggers signs of neurodegeneration including neurofibrillary tangles and amyloid deposits in the brain, whereas no cognitive changes were observed (Cox et al. 2016). BMAA is considered to have a low neurotoxic potency in adult rodents (Perry et al. 1989; Cruz-Aguado et al. 2006). However, BMAA is a developmental neurotoxin inducing long-term cognitive deficits as well as neurodegeneration, astrogliosis, and intracellular fibril formation in the hippocampus of adult rodents following neonatal exposure to BMAA (Karlsson et al. 2009b, 2014). BMAA also induces behavioral changes in neonates and perturbs serum metabolites that are associated with changes in energy metabolism and amino acid metabolism (Karlsson et al. 2009a; Engskog et al. 2013). The neonatal rat model is so far the only animal model that displays significant biochemical and behavioral effects after a low short-term dose of BMAA.

In the presence of bicarbonate ions BMAA forms a BMAA carbamate resembling glutamate and *N*-methyl-d-aspartic acid (NMDA) (Nunn and O’Brien 1989). Previous studies demonstrated that BMAA is a mixed glutamate receptor agonist that can induce excitotoxicity in primary neurons in vitro and in vivo (Weiss et al. 1989; Zeevalk and Nicklas 1989; Chiu et al. 2012) but BMAA is also suggested to induce neurotoxicity through other mechanisms such as oxidative stress (Lobner et al. 2014). Recent studies have demonstrated enrichment of proteins implicated in protein aggregation, and an increased protein ubiquitination in the adult hippocampus following neonatal administration suggesting BMAA-induced perturbations of cellular protein homeostasis (Karlsson et al. 2014). In addition, a dose-dependent increase of protein-associated BMAA in neonatal rat brain, especially in the hippocampus, has been observed (Karlsson et al. 2015). Other studies have suggested BMAA to be misincorporated into human proteins in exchange of l-serine causing protein aggregation in a cultured human fibroblast cell line (Dunlop et al. 2013; Glover et al. 2014). However, no incorporation of BMAA in proteins of proliferating human SH-SY5Y cells or in bacteria has been reported (Okle et al. 2013; van Onselen et al. 2015).

The perturbations preceding BMAA-induced long-term neurodegeneration in mammalian brain remain to be elucidated to get a better understanding of the underlying pathology. A possible route to gain valuable information is global metabolite profiling which is a powerful approach to perform a comprehensive analysis of small endogenous metabolites, i.e. the metabolome (Nicholson et al. 1999; Fiehn 2002; Kell 2004; Nicholson and Lindon 2008). This provides a biologically relevant molecular readout of the status of the cells reflecting phenotype, as well as the connectivity between active pathways (León et al. 2013; Gika et al. 2014). This information can be used to build a hypothesis related to the response. It is important to note that the resulting hypothesis only becomes relevant if the generated data is analytically reliable, thus numerous principles for quality assurance should be employed (Engskog et al. 2016; Vorkas et al. 2015). The analytical techniques of choice for detection of small metabolites in complex biological matrices is either nuclear magnetic resonance (NMR) spectroscopy or high resolution mass spectrometry (HRMS) or a combination of both to ensure a wider metabolome coverage (Lindon and Nicholson 2008; Zhang et al. 2010, 2013; Theodoridis et al. 2012; Vuckovic 2012).

The objective of the study presented here was to unravel early effects of BMAA on intracellular metabolites in a human neuroblastoma cell line utilizing a dual analytical platform profiling approach (NMR and UPLC-HRMS) focusing on low molecular weight endogenous metabolites of a polar nature. We employed a retinoic acid-differentiated SH-SY5Y cell line that has a limited proliferation rate and lacks functionally active NMDA receptors (Jantas et al. 2008) to examine BMAA-induced metabolic adaptations that are not related to excitotoxicity or oxidative stress. The unbiased and detailed metabolite profiling revealed a plentitude of experimentally reliable polar intracellular metabolite alterations. In particular, these alterations relate to perturbations of protein biosynthesis, amino acid metabolism pathways and citrate cycle that may interfere with fundamental metabolic pathways related to neurotransmission and contribute to long-term neurodegenerative changes.

## Materials and methods

### Chemicals

β-*N*-Methylamino-l-alanine, chloroform (analytical grade), ammonium formate (LC–MS grade), formic acid (99%, LC–MS grade), 2,2-dimethyl-2-silapentane-5-sulfonate sodium salt (DSS) and deuterium oxide (D_2_O, 99% D) were obtained from Sigma-Aldrich (Steinheim, Germany). Na_2_HPO_4_ and NaH_2_PO_4_ (analytical grade) were from Fluka (Buchs, Switzerland) while methanol (HPLC grade) and acetonitrile (LC–MS grade) were purchased from Fisher Scientific (Zurich, Switzerland). The water was purified using a Milli-Q™ Water system from MilliPore (Bedford, MA, USA).

### Cell culture and differentiation

SH-SY5Y neuroblastoma cells, passage number 21–24 (ECACC, Public Health England, UK) were cultured at 37 °C, in 5% CO_2_ in Eagle’s minimum essential medium (MEM; Life Technologies, Paisley, UK). The culture medium was supplemented with 10% fetal bovine serum (FBS; SVA, Uppsala, Sweden), 100 U/ml penicillin and 100 μg/ml streptomycin (Life Technologies, Paisley, UK). Cells were seeded in 10 cm cell culture plates (Sarstedt, Helsingborg, Sweden), allowed to settle for 72 h and then differentiated according to protocol previously reported (Dodurga et al. 2013), with some modifications. Briefly, the culture medium was replaced with a differentiation medium containing reduced serum concentration (2% FBS) containing 100 U/ml penicillin, 100 μg/ml streptomycin and 10 μM retinoic acid (Sigma-Aldrich, Steinheim, Germany). The differentiation medium was changed every other day for seven days. We exposed SH-SY5Y cells to BMAA in the presence of fetal calf serum to avoid activation of a stress response requiring adaptation to oxidative and metabolic stress (Harding et al. 2003; Valbuena et al. 2015) and to ensure the supply of amino acids for protein and glutathione synthesis. To generate biological replicates for metabolomics study, the neuroblastoma cells were cultured and differentiated in four different batches from different passages. As such, each batch consisted of two technical replicate untreated control cell plates and three cell plates treated with a low (50 µM), medium (250 µM) or high (1000 µM) concentration of BMAA (dissolved in the differentiation medium) for 24 h, respectively.

To verify that the cells had matured into a more neuron-like phenotype during differentiation, the expression of the neuronal markers βIII-tubulin and microtubule-associated protein-2 (MAP2) (Zeevalk and Nicklas 1989; Kovalevich and Langford 2013), was examined by immunocytochemistry. Cells were cultured and differentiated on polylysine coated glass in 24-well plates as mentioned above. After seven days of differentiation, the cells were exposed to BMAA (1000 µM; dissolved in the differentiation medium) for 24 h. Then the cells were fixated with 4% of paraformaldehyde for 15 min and washed with phosphate buffered saline (PBS, SVA, Uppsala, Sweden). To prevent non-specific binding the cells were incubated in 1.25% horse serum in 0.15% PBS-Triton X-100 (v/v). The cells were subsequently incubated with rabbit anti-β III tubulin (Covance, PRB-435P dilution 1:250) and mouse anti-microtubule associated protein 2 (MAP2) (Abcam, ab11267, dilution 1:250) overnight. The cells were then incubated with Alexa Fluor-488-conjugated goat anti-rabbit antibody and Alexa-Fluor-555-conjugated goat anti-mouse (Invitrogen, Carlsbad, CA, USA) and cell nuclei were stained by 4′,6-diamidino-2-phenylindole (Sigma-Aldrich, Steinheim, Germany).

### Cell viability

Cell viability was measured by MTT assay, as described elsewhere (Mosmann 1983), with some modifications. Briefly, SH-SY5Y cells were cultured and differentiated as mentioned above. After seven days of differentiation the cells were exposed to L-BMAA (250, 500 and 1000 µM; dissolved in the differentiation medium) for 24 h. Hydrogen peroxide (0.1 mM) was used as positive control. After exposure, the cells were treated with 5 mg/ml 3-(4,5-dimethylthiazol-2-yl)-2,5-diphenyl tetrazolium bromide (Sigma-Aldrich, Steinheim, Germany) for 4 h in 37 °C and then lysed with 0.7% sodium dodecylsulphate in isopropanol. Absorbance was measured at 570 nm in a FLUOstar Omega plate reader (BMG labtech, Offenburg, Germany) and cell viability was calculated as percent viability relative to the untreated control cells. The experiment was done in technical triplicates and repeated three times (nine samples total).

Oxidative stress was measured using the probe dichlorodihydrofluorescein as described elsewhere (LeBel et al. 1992; Okle et al. 2013), with some modifications. Briefly, 5 μM dichlorodihydrofluorescein diacetate (Invitrogen, Eugene Oregon, USA) in PBS was added to the differentiated cells for 30 min followed by exposure to L-BMAA (10, 100, 500 and 1000 µM; dissolved in the differentiation medium) for 45 min to detect formation of reactive oxygen species (ROS). Hydrogen peroxide (0.1 mM) was used as positive control. Fluorescence was measured at *λ*_ex/em_ 485/520 in a PolarStar Optima plate reader (BMG Labtech, Offenburg, Germany) and oxidative stress was calculated as percentage relative to the untreated control cells. The experiment was done in technical quadruplicates and repeated three times (12 samples total).

### Cell sample harvesting

Following incubation of cells for 24 h with BMAA dissolved in the differentiation medium or incubation of cells in the differentiation medium only (untreated control cells) the cells were harvested at approximately 95% confluence using cold sterile deionized water (MilliQ) as described previously (Engskog et al. 2015). All cell sample harvesting was performed on ice. The differentiation medium was removed and cells were rapidly washed three times with cold PBS followed by detachment of cells using a rubber-tipped cell scraper. The detached cells were collected in cold MilliQ water (1.4 ml), transferred to polypropylene tubes and snap-frozen in liquid N_2_ followed by thawing at 37 °C for 10 min. The freeze–thaw cycle was then repeated once with subsequent sonication on ice for 30 s. Cell samples were stored at −80 °C until intermediary metabolite extraction.

### Small polar cellular metabolite extraction

Harvested cells were thawed at room temperature and subjected to centrifugation (10 min, 2200 RCF) to remove precipitated cellular debris. For analysis with both NMR and LC–MS, a quality control (QC) sample was created by pooling an equal volume from all cell extracts. A fixed volume (1.7 ml) of supernatant (MilliQ) was transferred to a fresh extraction tube, followed by addition of chloroform and methanol resulting in the fixed proportions of 4:4:2.85 (MeOH:CHCl_3_:MilliQ) (León et al. 2013). The resulting two-phase system was gently vortexed and then left at 4 °C for 30 min before centrifugation (2200 RCF, 20 min, 4 °C). The aqueous phase was split for NMR (2.5 ml) and LC–MS (1.0 ml) analysis and transferred to new tubes, which were evaporated under N_2_ at 40 °C until dryness. The samples for NMR analysis were subsequently reconstituted in 0.7 ml phosphate buffered D_2_O (150 mM, pD 7) containing 2,2-dimethyl-2-silapentane-5-sulfonate sodium salt (DSS, 34 µM) as a chemical shift reference. The fractions recovered for LC–MS analysis were re-dissolved in acetonitrile:MilliQ (95:5) and transferred to sample vials.

### LC–MS analysis

Analysis was performed using an UHPLC system (ACQUITY UPLC I-Class, Waters) coupled to a Synapt G2S Q-TOF (Waters, Manchester, UK). All systems were controlled by MassLynx (Waters, version 4.1). Injection order was randomized, each sample being injected as a triplicate on a HILIC-amide, 1.7 μM, 55 mm × 2.1 mm I.D. column (Waters). The mobile phases consisted of A = 95:5:0.1 acetonitrile:10 mM ammonium formate:formic acid (v:v:v) and B = 50:50:0.1 acetonitrile: 10 mM ammonium formate:formic acid (v:v:v) with a gradient elution running from 100% A to 100% B over 18 min including 3 min column re-equilibration. The flow-rate was 0.4 ml/min and the sample injection volume was 5 μl.

MS-analysis was performed in resolution mode with MS^E^ using both ESI negative and positive ionization. The scan range was from *m*/*z* 50 to 1200 and argon was used as collision gas at a pressure of 3 × 10^−3^ bar. For MS-analysis the following parameters were used: capillary voltage of 1 kV (positive) and 2 kV (negative), cone voltage of 30 V, source temperature of 120 °C, desolvation temperature of 500 °C with nitrogen as desolvation and cone gas at flow-rates of 800 and 50 l/h, respectively. A collision energy ramp from 20 to 45 eV was used for MS^E^ acquisition. The instrument was calibrated using a 0.5 mM sodium formate solution in 2-propanol:water (90:10 v/v). Lock-mass correction was performed using a solution of 2 ng/μl leucine-enkephalin in acetonitrile:0.1% formic acid in water (50:50 v/v). Stable signal intensity, mass accuracy and retention time were monitored by repeated injections of the matrix (QC sample) to ensure a stabile system (Want et al. 2010; Vorkas et al. 2015; Engskog et al. 2016). Moreover, the QC sample was injected in triplicates in regular intervals throughout the analytical run to assess repeatability and overall system performance across the analytical batch (Want et al. 2010; Engskog et al. 2016).

### Data processing for LC–MS analysis

The raw LC–MS data was converted to NetCDF files by the DataBridge software (Masslynx version 4.1) and subjected to XCMS for peak detection and retention time alignment (Smith et al. 2006). The parameters in XCMS were set as follows: feature detection using the centWave function with Δ *m*/*z* of 8 ppm, minimum peak width of 5 s, maximum peak width of 25 s and signal to noise threshold of 10; grouping was performed with the standard group argument with mzwid = 0.05, retention time correction was performed using the obiwarp function. Experimental reproducibility was measured by determination of the coefficients of variation (CV) for each feature observed from the QC samples, with subsequent averaging of the CVs across the whole spectrum (Want et al. 2010; Vorkas et al. 2015). Moreover, features with a retention time below 45 s were not included as they eluted too close to the system void volume.

### Feature identification for LC–MS analysis

Feature identification was performed based on database searches against the Human Metabolome Database (V 3.0) (Wishart et al. 2013) and an in-house database with a molecular weight tolerance of ±0.02 Da, as well as examination of the corresponding MS/MS fragmentation obtained from MS^E^. Moreover, the processed data was subjected to isotope, adduct and fragmentation annotation by the aid of the R-based addition to XCMS referred to as CAMERA (Kuhl et al. 2012). The metabolites identified should be seen as putatively annotated compounds (based upon physicochemical properties and/or spectral similarity) according to the Metabolomics Standards Initiative nomenclature (Sumner et al. 2007; Creek et al. 2014).

### NMR spectroscopy

Nuclear magnetic resonance measurements were carried out at 298 K on a Bruker Avance 600 MHz (Bruker BioSpin GmbH, Rheinstetten, Germany) equipped with a cryoprobe. For each sample, the 1D NOESYPR1D standard pulse sequence (–RD-90°-*t*_1_-90°-*t*_m_-90°-ACQ) was used. Each pulse had a 90° pulse length; the total amount of FIDs recorded were 256 collected into 64 K data points which was zero-filled to 128 K data points. The spectra width was set to 7183.91 Hz giving a spectral acquisition time of 4.56 s. The *t*_1_ and *t*_m_ was set to 6 µs and 180 ms, respectively, and relaxation delay (D1) was 3 s, resulting in a total acquisition time of 33 min. The 1D spectra were manually phased and baseline corrected and the ^1^H chemical shifts were referenced to added DSS using the ACDLABS (version 12.01, Advanced Chemistry Development, Inc, Canada).

Each ^1^H NMR spectrum, within a range of 0–10 ppm, was reduced to 873 bins of fixed width (0.01 ppm) excluding resonance regions for water (*δ* 5.15–4.67 ppm) and the internal standard (DSS, *δ* 0.65–0.00, 1.77–1.72 and 2.92–2.88 ppm). The signal intensity in each bin was integrated using ACDLABS. Data were imported to Microsoft Excel (Microsoft Office 2007, Redmond, WA, USA) and normalized to unit total intensity. Assignments of NMR peaks were performed according to the Metabolomics Standards Initiative (Sumner et al. 2007; Creek et al. 2014) with the aid of the Human Metabolome Database (V 3.0) (Wishart et al. 2013).

### Univariate and multivariate data analysis

Positive and negative LC–MS data as well as NMR data were normalized to total intensity in Microsoft Excel and separately analyzed by multivariate data analysis using the SIMCA-P+ (version 13.0, Umetrics, Umeå, Sweden) computational software package. A criterion of CV <30% was used to afford analytically reliable features for multivariate and univariate analysis (Bijlsma et al. 2006; Dunn et al. 2012; Vorkas et al. 2015; Engskog et al. 2016). Data were Pareto scaled, as implemented in the SIMCA-P+ package, prior to further analysis. Principal component analysis (PCA) and orthogonal projection to latent structures discriminant analysis (OPLS-DA) in combination with visualization by S-plots were used for identification of key differences between groups (Trygg and Wold 2002; Trygg et al. 2007). The OPLS models were evaluated based on the model’s prediction *Q* ^2^ values, only models with *Q* ^2^ values >0.4 were considered relevant. Spectral bins and features were selected from the reliable models based on the following criteria: *p*_corr_ ≥ 0.5 for both HILIC-MS data and NMR data. All spectral bins and feature which met these criteria were also subjected to univariate analysis with one-way analysis of variance (ANOVA) with post hoc Tukey’s tests using the web-based software Metaboanalyst (Xia et al. 2015). Features and bins, which resulted in a *p* value <0.05 and were significant according to the Tukey’s post hoc test were retained for further analysis.

### Pathway analysis based on NMR and LC–MS data

The significantly altered cellular metabolites obtained from NMR and LC–MS analysis (each concentration vs. control separately) were subjected to pathway analysis using Metaboanalyst which is based on KEGG metabolic pathways. Pathway analysis was conducted using the hypergeometric algorithm for over-representation analysis (ORA) and the relative-betweenness centrality algorithm for pathway topology analysis. A *p* value <0.05 and false discovery rate (FDR) <5% were considered significant for pathway analysis.

## Results

### Cell differentiation and viability

**Fig. 1:**
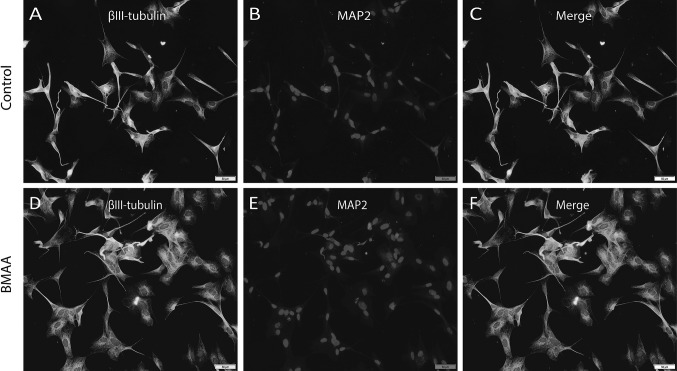
Double-antigen immunofluorescence microscopic images for neuronal specific marker expression of βIII-tubulin and MAP2 in untreated control cells (**a**–**c**) and BMAA-treated (1000 µM) differentiated SH-SY5Y human cells for 24 h (**d**–**f**). Representative fields of immunostained cells reveal no major changes in neuronal specific marker expression or cell density in BMAA-treated cells as compared to untreated control cells. Expression of βIII-tubulin is shown in green (**a**, **d**) and expression of MAP2 is shown in *red* (**b**, **e**). Nuclei staining with DAPI is shown in *blue*. *Scale bar* 50 μm (**a**–**f**). Magnification ×20 (color figure online)

**Fig. 2:**
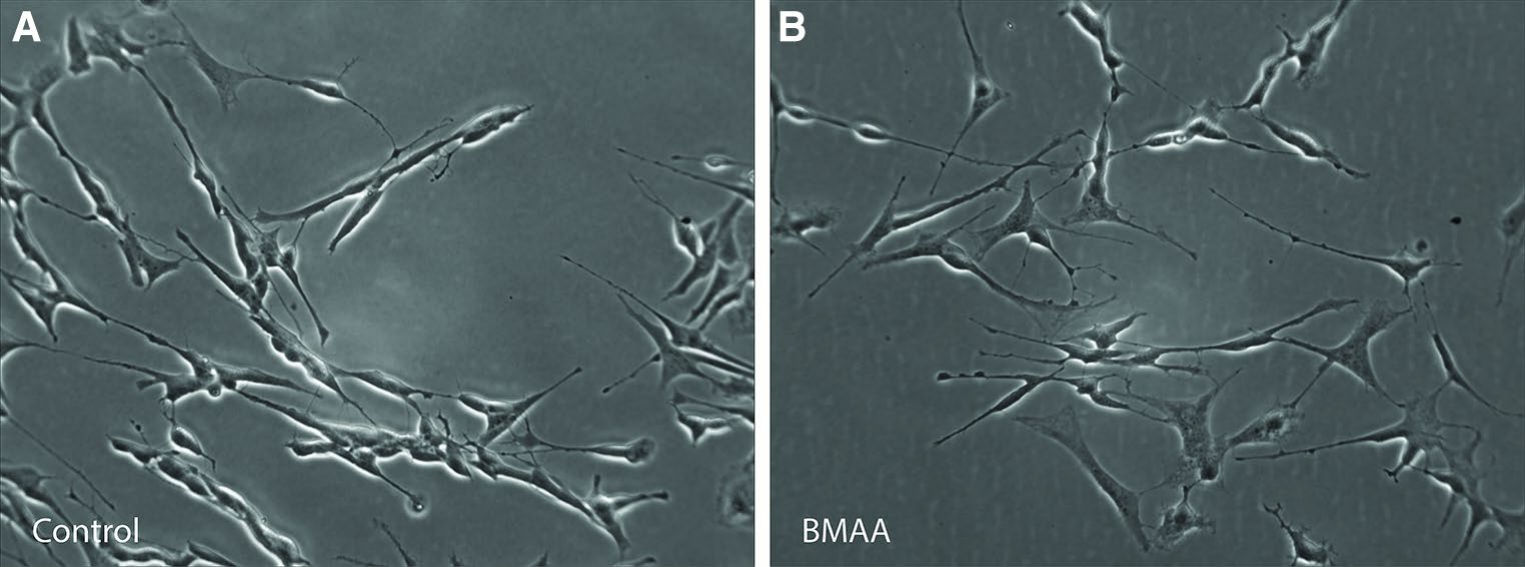
Phase contrast images of untreated control cells (**a**) and BMAA (1000 µM) treated differentiated SH-SY5Y cells (**b**) for 24 h. Representative fields of cells reveal no major changes in morphology or in cell density in BMAA-exposed cells compared to untreated control cells. Magnification ×20

Seven days after treatment with retinoic acid the SH-SY5Y cells had differentiated into a neuron-like phenotype and exhibited characteristic morphology including extensive neurite outgrowth. In addition, the expression of neuron specific protein markers β III tubulin and MAP2 in the differentiated SH-SY5Y cells were detected by immunofluorescence. Following incubation with BMAA (1000 µM) for 24 h there were no major changes in cell density, neuron specific protein expression or neurite outgrowth as compared to untreated control cells (Figs. 1, 2). Furthermore, after incubation with BMAA (up to 1000 µM) for 24 h the cells showed no significant change in viability or oxidative stress as compared to untreated control cells (data not shown).

### NMR spectroscopy profiling

**Fig. 3:**
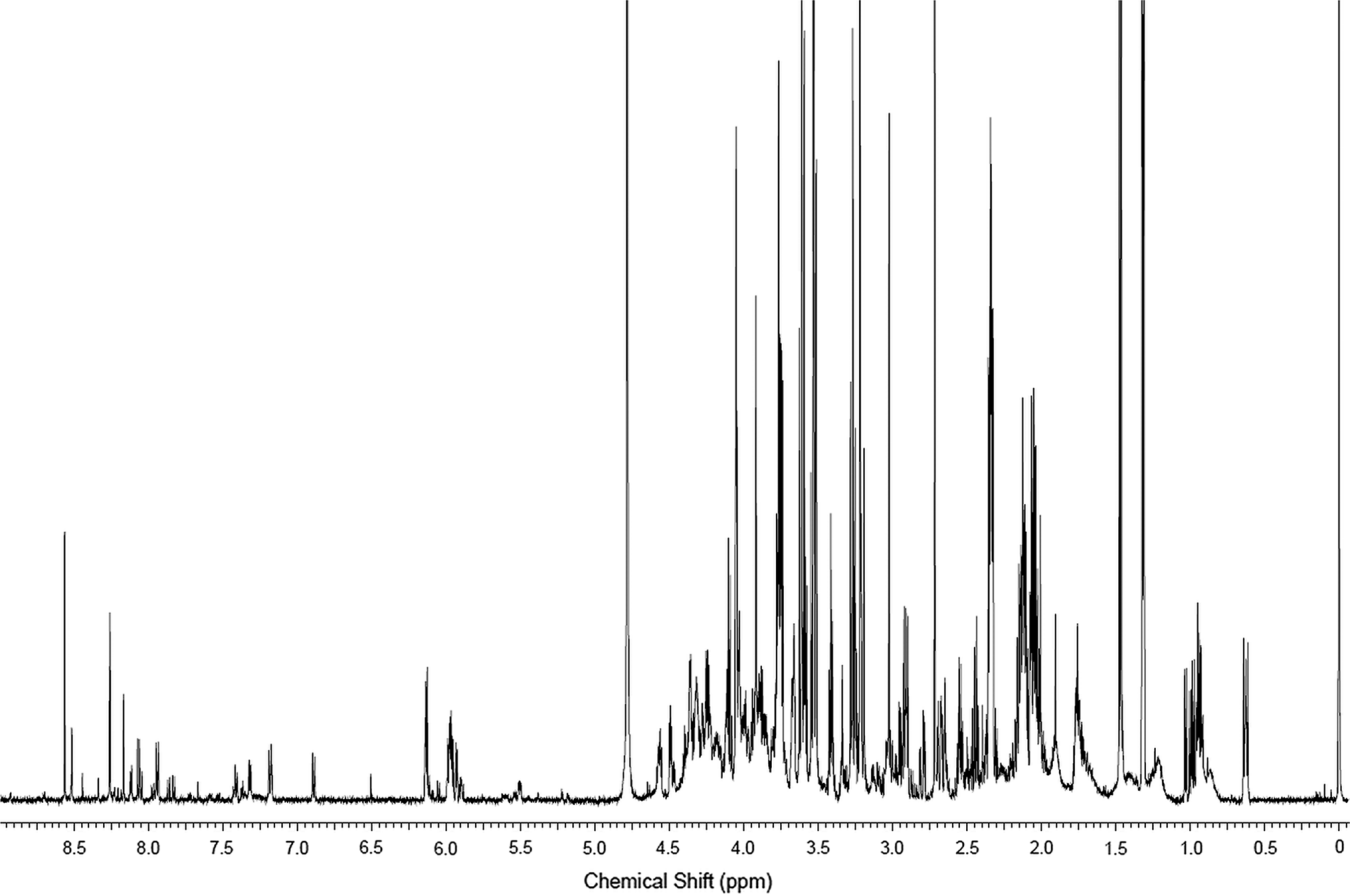
Representative NMR spectra obtained from the pooled QC sample dissolved in 0.7 ml phosphate buffered D_2_O (150 mM, pD 7). Chemical shifts are expressed relative to the DSS resonance set at 0.00 ppm

The profiling experiments based on NMR-spectroscopy resulted in information rich spectra, i.e. containing a plentitude of signals of varying intensity; an example is given in Fig. 3. In general, all spectra were of high intensity. However, this also results in severe overlap in certain spectral regions (see for example region at *δ* 4.5–2.0 p.p.m in Fig. 3). Consequently, unambiguous metabolite identifications were elusive, but some plausible annotations are presented at the end of this section. Generated OPLS-DA models demonstrated adequate group differentiation for the high (1.0 mM) and medium (250 µM) BMAA concentration samples as compared to untreated control cells. However, the experiments using a low BMAA (50 µM) concentration resulted in models with a poor predictive power (*Q* ^2^ < 0.4). Spectral bins were selected from S-plots according to the criteria described in materials and methods; these spectral bins were subsequently tested for significance by ANOVA with parametric post hoc Tukey’s tests. Briefly, forty-six spectral bins were significantly altered of which only a few could be tentatively assigned due to overlap of resonances. Briefly, following exposure to the high concentration of BMAA there was an enrichment of the following tentatively assigned metabolites: l-aspartate, taurine and l-serine. Moreover, following exposure to the medium concentration of BMAA there was an enrichment of the following tentatively assigned metabolites: hypotaurine, taurine and histamine, whereas cytidine monophosphate was reduced.

### LC–MS profiling

A plentitude of features were detected based on data acquired in negative and positive ionization mode. To arrive at analytically reliable results, QC data was evaluated by univariate analysis to only retain features common to the study specific samples with a retention time above 45 s and CV values below 30% as previously described. As such, this filtering procedure retained 1168 features in positive ionization and 1593 features based on data acquired in negative ionization mode. It is important to state that these numbers do not represent the number of detected metabolites as each metabolite may generate several features representing isotopes, fragments and adducts originating from the same compound. All features were normalized to total intensity and were subsequently imported into SIMCA for multivariate analysis. An overview of the results based on these selection principles are presented in Table 1.

**Table 1:** Overview of selection criteria based on LC–MS analysis in positive and negative ionization mode

Stable features^a^	LC–MS (positive)			LC–MS (negative)		
	1168			1593		
	High dose	Medium dose	Low dose	High dose	Medium dose	Low dose
OPLS-DA *Q* ^2^ value	0.91	0.93	0.53	0.94	0.89	0.52
Altered features^b^	568	389	339	435	306	115
Significant metabolites^c^	28	27	1	30	13	1

^a^Retention time >45 s, CV <30%

^b^0.5 < *p*_corr_ < −0.5

^c^Annotated metabolites (*M*_W_ < 700 Da, significant according to the post hoc Tukey’s test)

**Fig. 4:**
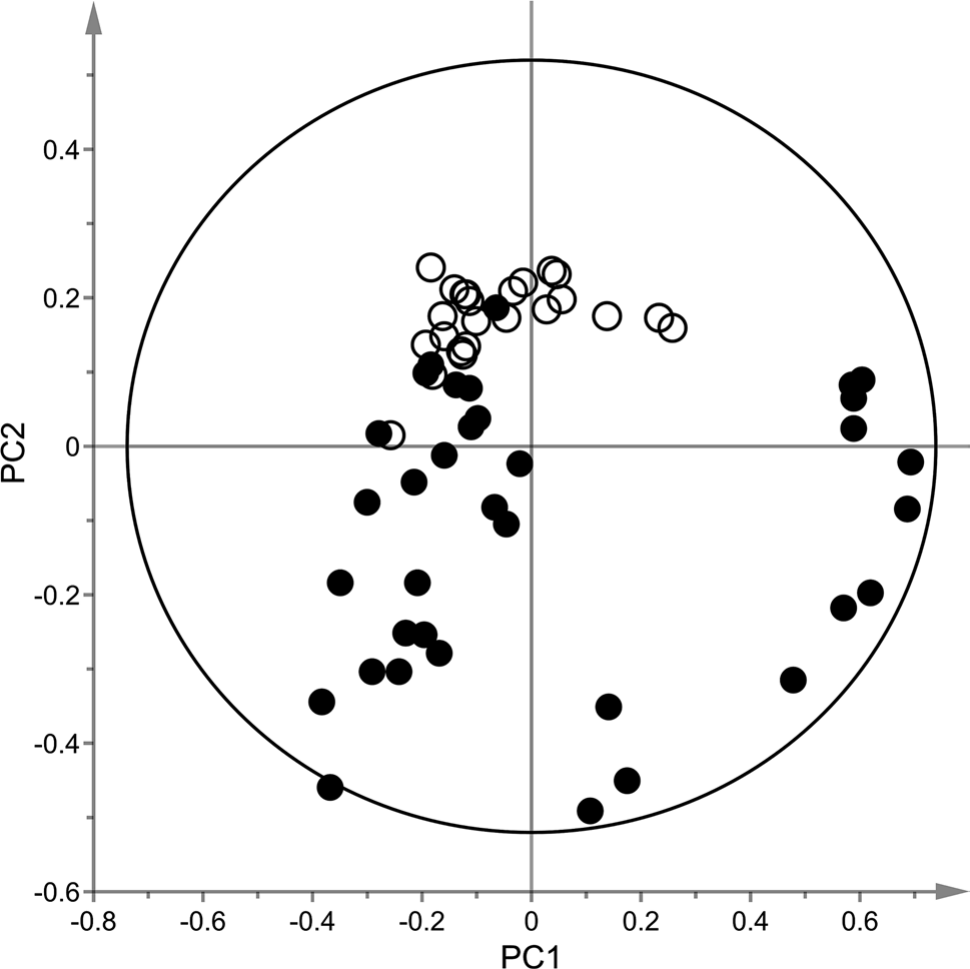
PCA model (*R* ^2^ *X* = 0.79, *Q* ^2^ *X* = 0.63) displaying untreated control cells (*open circles*) vs BMAA exposed cells (low (50 µM), medium (250 µM) and high (1000 µM) concentration, *filled circles*) obtained from LC–MS analysis in positive mode. Data were pre-processed with XCMS, normalized to total signal intensity and subjected to Pareto scaling using the SIMCA-P+ software. Moreover, only features with CV values below 30% were included in the analysis

**Fig. 5:**
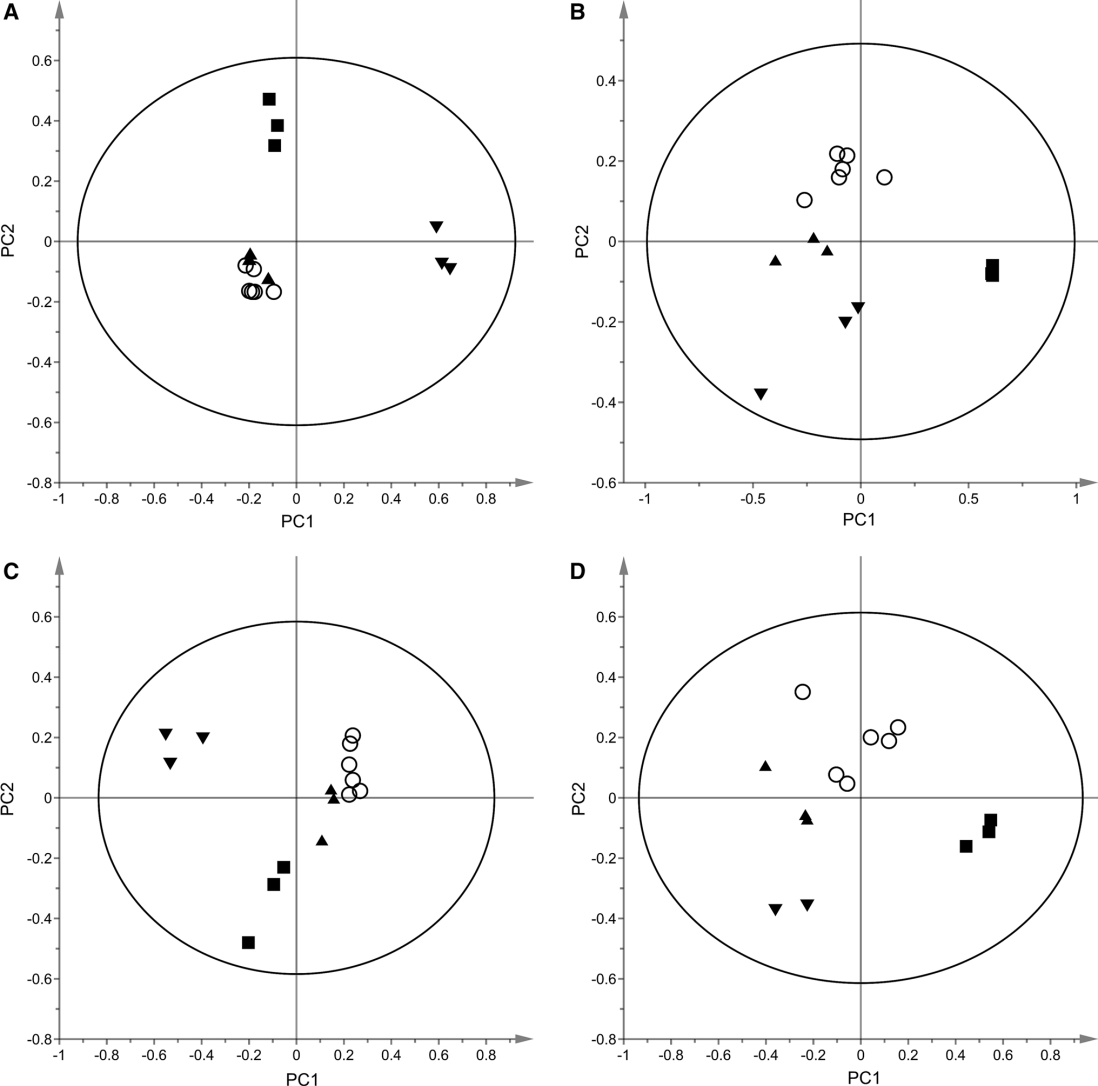
PCA models of the four biological batches (**a** *R* ^2^ *X* = 0.73, *Q* ^2^ *X* = 0.63, **b** *R* ^2^ *X* = 0.78, *Q* ^2^ *X* = 0.58, **c** *R* ^2^ *X* = 0.78, *Q* ^2^ *X* = 0.53, **d** *R* ^2^ *X* = 0.84, *Q* ^2^ *X* = 0.64) displaying untreated control cell (*open circles*) samples, high (1000 µM) BMAA concentration (*filled inverted triangles*) samples, medium (500 µM) BMAA concentration (*filled squares*) samples and low (50 µM) BMAA concentration (*filled upright triangles*) samples obtained from LC–MS analysis in positive ionization mode. Each sample was injected three times in a random order throughout the analytical run. Data were pre-processed with XCMS, normalized to total signal intensity and subjected to Pareto scaling using the SIMCA-P+ software. Moreover, only features with CV values below 30% were included in the analysis

Multivariate modeling with PCA illustrates the complexity of biological variance observed in the data based on different passages of cells (Fig. 4). As seen, most samples originating from BMAA-exposed cells are found in the lower right and left side of the plot (Fig. 4, filled circles) while untreated control cell samples (Fig. 4, open circles) are found in the upper region which indicate an altered metabolome. Furthermore, all low concentration BMAA samples are found in close proximity to the untreated control cell samples demonstrating a lesser effect due to exposure. It is plausible to assume that a higher BMAA concentration generates a larger disturbance in the metabolome as compared to low BMAA concentration as shown in Fig. 4. In addition, this trend is consistent in data from negative ionization (data not shown). Moreover, samples also cluster according to the batch (passage) of the cells; hence a large contribution is observed which from now on will be referred to as inter-batch variation. To more adequately display the relevant difference according to BMAA concentration, representative examples of a PCA plots for each batch is given in Fig. 5a–d. As observed, SH-SY5Y cells treated with a medium (Fig. 5, filled squares) or high BMAA concentration (Fig. 5, filled inverted triangles) results in a more drastic alteration of the metabolome as compared to a low BMAA concentration (Fig. 5, filled upright triangles) and untreated control cells (Fig. 5, open circles). To study the metabolic alterations caused by BMAA in a concentration-dependent manner, OPLS-DA models were created for each treatment as compared to vehicle; all models gave sufficient predictive *Q* ^2^ values (>0.5). Features that were important to describe the difference between vehicle and BMAA exposed cells were selected from the corresponding S-plots according to the criteria stated previously. These features were subsequently tested for significance with ANOVA with post hoc Tukey’s tests after which differentiating metabolites were annotated for each BMAA concentration group as compared to vehicle (Table 2). The annotated metabolites belong to a diverse set of metabolite classes, including numerous amino acids and their derivates as well as carnitines, nucleosides and smaller organic acids. No traces of the masses corresponding to the parent compound BMAA and known hepatic BMAA metabolites (methylamine, 2,3-diaminopropanoic acid) (Nunn and Ponnusamy 2009) were found upon examination of the raw data. The recorded data for the high BMAA concentration experiment is similar to the response observed in the medium and low BMAA concentration experiments as compared to vehicle (data not shown). As the response to BMAA exposure was readily observed in the high BMAA concentration, further pathway analysis focused upon this exposure.

**Table 2:** List of significant metabolite differences observed between untreated control cells and BMAA exposed human differentiated SH-SY5Y cells [high (1000 µM), medium (250 µM) and low (50 µM) BMAA concentrations]

Annotation	Alteration	ANOVA (*p* value)^a^	Tukey test			Fold change^b^	Metabolite class
			H	M	L		
Gamma aminobutyrate	↑	1.2*E*−05	×			120	Organic acid
Glutamate gamma-semialdehyde	↑	1.2*E*−06	×			39	Organic acid
5-Hydroxykynurenamine	↑	1.8*E*−05	×			36	Amine
*N*-Acetyl-glutamate 5-semialdehyde	↑	2.9*E*−05	×			16	Amino acid derivate
Biotin	↑	8.1*E*−05	×			11	Vitamin
5′-Methylthioadenosine	↑	1.6*E*−04	×	×		7.4	Nucleoside
Citrate	↑	5.8*E*−06	×	×		7.0	Organic acid
Pyruvate	↑	4.0*E*−07	×	×		6.9	Organic acid
Tryptophan	↑	2.6*E*−05	×	×		6.6	Amino acid
l-3-Hydroxykynurenine	↑	4.5*E*−06	×	×		6.5	Amine
*N*-Acetylhistidine	↑	2.2*E*−06	×			6.4	Amino acid derivate
Cytidine	↑	4.7*E*−04	×			5.9	Nucleoside
d-4′-Phosphopantothenate	↑	1.6*E*−06	×			5.8	Organic acid
Guanine	↑	4.4*E*−04		×		5.7	Purine
Diguanosine diphosphate	↑	7.9*E*−05	×	×		5.7	Nucleotide
Glutamate	↑	1.3*E*−06	×	×		5.5	Amino acid
Phenylalanine	↑	6.3*E*−06	×			5.5	Amino acid
Argininosuccinate	↑	2.0*E*−05	×	×		5.4	Amino acid derivate
*N*-Acetyl-aspartate	↑	4.6*E*−05	×	×		5.3	Amino acid derivate
*N*-Formylmethionine	↑	4.8*E*−05	×	×		5.3	Amino acid derivate
*S*-Acetyldihydrolipoamide	↑	1.2*E*−05	×	×		5.2	Lipid
Spermic acid	↑	9.1*E*−06	×	×		5.0	Organic acid
2-Ketoglutaramate	↑	7.2*E*−07	×	×		4.9	Amino acid derivate
*N*-a-Acetylcitrulline	↑	2.2*E*−06	×			4.9	Organic acid
l-Acetylcarnitine	↑	2.6*E*−07	×	×		4.9	Carnitine
*N*-Acetyl-glucosamine	↑	1.0*E*−04	×	×		4.8	Carbohydrate
Butyrylcarnitine	↑	1.4*E*−05	×	×		4.8	Carnitine
Guanosine	↑	2.3*E*−05	×			4.8	Nucleoside
Thiamine	↑	4.6*E*−06	×	×		4.7	Vitamin
l-Malate	↑	3.5*E*−04		×		4.6	Organic acid
Acetylcysteine	↑	1.4*E*−04	×	×		4.5	Amino acid derivate
Leucine/isoleucine	↑	1.3*E*−05	×			4.5	Amino acid
Threonine	↑	2.6*E*−04	×			4.3	Amino acid
1-Methylinosine	↑	6.7*E*−05	×			4.3	Nucleoside
Proline	↑	7.0*E*−03		×		4.2	Amino acid
Dihydrothymine	↑	7.6*E*−04	×			4.0	Pyrimidone
Propionylcarnitine	↑	7.1*E*−05	×	×		4.0	Carnitine
*N*-Ribosylhistidine	↑	8.5*E*−06	×			3.9	Nucleoside
Pantothenate	↑	1.1*E*−05	×	×		3.9	Organic acid
Adenosine	↑	3.3*E*−05	×	×		3.9	Nucleoside
Inosine	↑	1.7*E*−05	×			3.3	Nucleoside
Pivaloylcarnitine	↑	1.2*E*−03	×	×		3.1	Carnitine
Choline (fragment)	↑	1.3*E*−04	×			3.0	Choline
Hypoxanthine	↑	2.0*E*−04	×			2.8	Purine
Uridine	↑	3.1*E*−05	×			2.6	Nucleoside
Phosphatidylethanolamine	↑	4.9*E*−04	×			2.0	Lipid
Taurine	↑	4.7*E*−02	×			1.7	Organic acid
Spermic acid 1	↓	1.9*E*−02		×		0.49	Organic acid
Spermine	↓	5.9*E*−03	×	×		0.41	Amine
3-Hydroxynonanoyl carnitine	↓	1.1*E*−02		×		0.41	Carnitine
2-Methylbutyroylcarnitine	↓	8.2*E*−03		×		0.39	Carnitine
Gamma-Aminobutyryl-lysine	↓	2.9*E*−02		×		0.39	Amino acid derivate
Tetradecanedioic acid	↓	3.7*E*−02		×		0.36	Lipid
Lysine	↓	1.2*E*−02		×		0.35	Amino acid
*N*-6-acetyl-lysine	↓	6.6*E*−04	×	×		0.25	Amino acid derivate
Homocysteinesulfinic acid	↓	1.6*E*−10	×	×	×	0.19	Organic acid
Biopterin	↓	4.7*E*−06	×	×		0.10	Pteridine

*L* low BMAA concentration, *M* medium BMAA concentration, *H* high BMAA concentration as compared to control

^a^The listed *p* values are the least significant values determined for the three doses (high, medium or low BMAA concentrations)

^b^The listed fold change values are the lowest values determined for the three doses (high, medium or low BMAA concentrations)

### Pathway analysis

The significantly altered and annotated metabolites obtained from LC–MS and NMR-spectroscopy profiling for the high BMAA concentration vs. vehicle were subjected to pathway analysis using the web-based software Metaboanalyst (Xia et al. 2015). An overview of the metabolite data indicated that there was a preferential change of the protein biosynthesis (*p* < 0.05) in the BMAA-treated SHSY-5Y cells and that two of the metabolites can be associated with the hippocampus: gamma aminobutyric acid (GABA) and *N*-acetylaspartate (NAA).

In the high BMAA concentration, alterations in seven metabolites (NAA, argininosuccinate, l-aspartate, pyruvate, 2-ketoglutaramate, l-glutamate and GABA) out of the 24 metabolites involved in alanine, aspartate and glutamate metabolism were observed (*p* < 2.15 × 10^−7^, FDR < 1.71 × 10^−5^). For the citrate cycle three metabolites (pyruvate, l-malate, citrate) of 20 metabolites were altered (*p* < 5.35 × 10^−4^, FDR < 0.02). For the arginine-proline metabolism eight metabolites (l-glutamate-gamma-semialdehyde, l-glutamate, argininosuccinate, *N*-acetyl-l-glutamate-5-semialdehyde, l-proline, spermine, GABA and pyruvate) of 77 metabolites were altered (*p* < 6.68 × 10^−4^, FDR < 0.02 × 10^−5^). No significant alterations of energy-related metabolites such as glucose, lactate and ATP were observed. In addition, enrichment of additional metabolites related to neurotransmission, e.g. taurine was observed.

## Discussion

The present study demonstrated that the non-proteinogenic neurotoxin BMAA induces significant alterations of polar intracellular metabolites in differentiated human SH-SY5Y neuroblastoma cells. However, it is important to state that untargeted metabolic profiling used in the present study will describe the status of the cells as a snapshot taken during the time-point of harvesting (24 h of exposure), thus not providing information related to flux throughout the biological system. Nevertheless, the quantitative measurements reported here facilitate further mechanistic studies on the effects of BMAA exposure in neuronal cells. It should be noted that BMAA did not decrease the cell viability or induced ROS production in the differentiated SH-SY5Y cells demonstrating that the observed alterations of intracellular metabolites were not due to acute excitotoxicity or oxidative stress. Pathway analysis using the web-based software Metaboanalyst (Xia et al. 2015) indicated that there was a preferential perturbation of the protein biosynthesis and that the BMAA-induced perturbations are typically associated with the hippocampus. The detailed metabolite profiling revealed significant perturbations in amino acid metabolism pathways involved in neurotransmission, preferentially in the alanine, aspartate and glutamate metabolism but also the arginine–proline metabolism. The perturbations of the amino acid metabolism pathways were not the result of single metabolite alterations but due to a combination of metabolite changes. Amino acids are mainly building blocks for proteins but other functions are also of importance. A number of amino acids such as β-alanine, aspartate, glutamate, GABA, proline, glycine, serine, and taurine serve as neurotransmitters and/or neuromodulators (Santos et al. 2012). The present study revealed that BMAA perturbed metabolism pathways involving several neurotransmitters/neuromodulators and/or their precursors suggesting that BMAA-induced neurodegenerative effects in rodents and vervet monkeys may not only involve a potential misincorporation of this unusual amino acid into proteins but also other important functions such as neurotransmission. Furthermore, the observed BMAA-induced effects in human neuronal-like cells are in analogy with our previous study in BMAA-treated neonatal rodents showing perturbations of serum metabolites associated with changes in energy and amino acid metabolism (Engskog et al. 2013).

### Perturbations of amino acid metabolism pathways

**Fig. 6:**
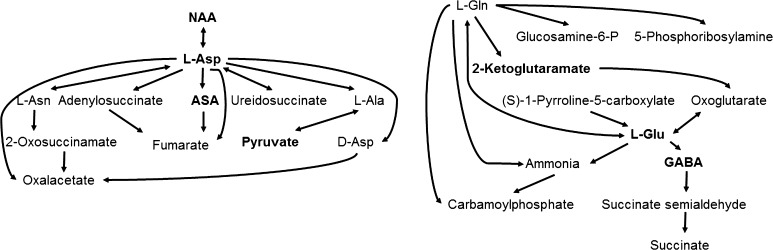
Overview of key altered metabolites found in differentiated SH-SY5Y human cells exposed to high level BMAA (1000 µM) as compared to untreated control cells. Main pathways involved are alanine, aspartate and glutamate metabolism. This map is a graphical adaptation based on pathway maps obtained from MetaboAnalyst (Xia et al. 2009), metabolites in bold are significantly upregulated as compared to vehicle. Also, see Table 2 for additional information. *NAA N*-acetylaspartate, *l* *-Asp* l-aspartate, *l* *-Asn* l-asparagine, *ASA* argininosuccinate, *l* *-Ala* l-alanine, *d* *-Asp* d-aspartate, *l* *-Gln* l-glutamine, *glucosamine-6-P* glucosamine-6-phosphate, *L-Glu* l-glutamate, *GABA* gamma aminobutyric acid

β-Methylamino-l-alanine is reported by Nunn and Ponnusamy (2009) to rapidly react nonenzymatically with pyridoxal-5′-phosphate in vitro. Pyridoxal-5′-phosphate is a cofactor in many biological reactions including transamination reactions of amino acids (Mozzarelli and Bettati 2006). The present study demonstrated that there was a preferential perturbation of the metabolism pathways of alanine, aspartate and glutamate in the human neuronal-like cells (Fig. 6). Aspartate and glutamate are known as major excitatory transmitters acting in the same way as BMAA on glutamatergic NMDA receptors inducing excitotoxicity in primary neurons (Rush et al. 2012). The biosynthesis of these amino acids is also connected with intermediates in the citrate cycle and a significant perturbation of the citrate cycle was also observed in the BMAA-treated cells. The present study also revealed a significant enrichment of biotin. This vitamin acts as a coenzyme for carboxylases that are involved in the production of key intermediates in the citrate cycle, which is essential for the energy production in neurons. However, there were no significant alterations of glucose, lactate and ATP in the BMAA-treated cells suggesting that the cellular energy supplies were maintained. In conclusion, these results demonstrate that the observed BMAA-induced perturbations of excitatory transmitter pathways in human neuronal-like cells are not related to acute excitotoxicity, oxidative stress or energy depletion but due to other metabolic adaptations.

The pyridoxal-5′-phosphate-dependent enzymes are known to catalyze a wide variety of biochemical reactions suggesting that BMAA may perturb many metabolic pathways as demonstrated in the present study. The dismutation of BMAA forming ammonium via the reaction with pyridoxal-5′-phosphate is proposed by Nunn and Ponnusamy (2009) to lead to an increased synthesis of alanine via transamination with glutamate. The present study did not reveal an intracellular enrichment of alanine in the BMAA-treated cells but there was a significant perturbation of the alanine metabolism. In contrast, there was an enrichment of intracellular glutamate, *N*-acetyl-l-glutamate 5-semialdehyde and glutamate-gamma-semialdehyde in the BMAA-exposed cells. Neurons cannot synthesize glutamate from glucose but transamination of alanine and aspartate is known to lead to glutamate (Sookoian and Pirola 2012). The BMAA-induced enrichment of glutamate in the differentiated SH-SY5Y cells could, therefore, be secondary to a transient enrichment of alanine and aspartate. The enrichment of glutamate-gamma-semialdehyde and *N*-acetyl-l-glutamate 5-semialdehyde is most likely secondary to the enrichment of the precursor glutamate. The present study also revealed a significant enrichment of the inhibitory transmitter GABA in the BMAA-treated cells. The enrichment of GABA may also be secondary to the enrichment of glutamate since glutamate is a precursor to GABA (Tranberg et al. 2004).

An enrichment of intracellular aspartate and NAA were also observed in the BMAA-treated SH-SY5Y cells. The neuron-specific NAA is mainly synthesized in neurons and is considered as a marker of neuronal viability (Tsang et al. 2009). NAA is normally catabolized in glial cells after NMDA-receptor mediated efflux from primary neurons (Tranberg et al. 2004). de Munck and coworkers (2013) recently reported that repeated administration of BMAA reduced the level of NAA in the spinal cord and cortex of adult rats. The differential effects of BMAA on NAA in cultured SH-SY5Y cells and in rodents are most likely related to the lack of functional NMDA receptors in the SH-SY5Y cells leading to a reduced efflux of NAA.

A significant perturbation of the arginine-proline metabolism pathways in the BMAA-treated cells was also observed. Arginine is involved in several metabolic processes and alterations may have the potential to disrupt many cellular functions. Arginine is for instance participating in the formation of nitric oxide (NO) and NO production in neurons which may induce neurotoxicity or be neuroprotective (Tripathy et al. 2015). However, no significant enrichment of arginine was observed in the BMAA-treated cells suggesting that there was no major changes in NO production. In contrast, there was a significant enrichment of proline, which is known to be derived from both arginine and glutamate. Since there was no enrichment of arginine, the enrichment of proline is probably related to the observed enrichment of glutamate.

The NMR studies demonstrated a tentative enrichment of serine in the BMAA-treated neuronal-like cells, although serine was not detected with LC–MS analysis. It is important to state that not all small molecules ionize with the same efficiency during the MS-analysis thus providing a plausible explanation to why serine was not detected either as [M+H], [M−H] or possible adducts (Engskog et al. 2016). However, there was a perturbation of the glycine, serine and threonine metabolism pathways in the BMAA-treated cells. This is of special interest due to recent observations by Dunlop and coworkers. They suggest that BMAA is misincorporated in place of serine into human proteins based on studies showing that the uptake and binding of radiolabelled BMAA to proteins in cultured fibroblasts are decreased by excess serine (Dunlop et al. 2013; Glover et al. 2014). Serine has also been reported to decrease BMAA-induced ER stress markers and caspase-3 activation in proliferating neuronal cells (Main et al. 2016) and co-administration of serine and BMAA reduced the density of neurofibrillary tangles in vervet monkeys (Cox et al. 2016). However, since the cellular uptake of BMAA is a transport-mediated process, the protective effects of serine on BMAA-induced toxicity may also be related to a decreased cellular uptake of BMAA. Preliminary studies suggest that BMAA interacts with the alanine–serine–cysteine and glutamine transporter ASCT2 in proliferating SH-SY5Y cells (Andersson et al. unpublished data). Other studies report that there was no evidence of incorporation of BMAA in newly synthesized proteins in proliferating SH-SY5Y cells or in bacteria (Okle et al. 2013; van Onselen et al. 2015). We have found that BMAA is protein-associated in the rodent brain, especially in the hippocampus (Karlsson et al. 2015). The protein-association in the brain was; however, considerably lower than that in the liver, which so far has not been reported to be a target organ for BMAA. Obviously, further studies on the effects of serine on BMAA-induced neurotoxicity in vivo and in vitro are needed.

Interestingly, the present study revealed an enrichment of taurine in BMAA-treated SH-SY5Y cells. Taurine plays an important role in regulating cell volume to prevent stress and is neuroprotective (El Idrissi et al. 2013). It resembles GABA in its chemical structure and is modulating neurotransmission, as well as acting as a partial agonist for glycine and GABA receptors. Nunn and Ponnusamy (2009) reported that taurine is reduced in BMAA-exposed rat brain slices. This is suggested to be due to an inhibition of taurine biosynthesis or to a BMAA-induced activation of AMPA and NMDA receptors in neurons leading to efflux of taurine (Oja and Saransaari 2013). Since there is a lack of functional NMDA receptors in SH SY5Y cells, an NMDA-activated efflux of taurine and hypotaurine in these cells is less likely to occur. This may lead to the observed intracellular enrichment of taurine in the BMAA-treated SH SY5Y cells (Tranberg et al. 2004).

### Metabolism of BMAA

Early experiments demonstrated that BMAA is converted by l-amino acid oxidase to the corresponding imino acid, which is hydrolyzed to *N*-methylaminopyruvate and ammonia. The keto acid can be readily oxidized to *N*-methylglycine (Hashmi and Anders 1991). In rodent brain, BMAA is suggested to be metabolized by CYP to formaldehyde (Kisby and Spencer 2011) and to methylamine and 2,3-diaminopropionic acid in kidney and liver homogenates (Nunn and Ponnusamy 2009). However, none of these potential BMAA metabolites were observed in the BMAA-treated SH-SY5Y cells. It is, however, important to state that several explanations are possible: (1) the metabolism of BMAA in human neuronal-like cells may be insignificant or different as compared to other tissues (2) the employed analytical techniques cannot detect potential BMAA metabolites (below detection limit, difficulties to ionize the substances or too low mass), (3) the potential BMAA metabolites are extensively metabolized in the cells or (4) the formed BMAA fragments/adducts are difficult to relate to the parent compound.

## Conclusions

The human exposure to the cyanobacterial toxin and non-proteinogenic amino acid BMAA is of concern as climate warming and eutrophication results in increasing cyanobacterial growth in water bodies across the world. The present study, combining metabolic profiling and multivariate pattern recognition techniques, demonstrated that BMAA perturbed the protein biosynthesis, the amino acid metabolism pathways and the citrate cycle in differentiated human SH-SY5Y neuroblastoma cells. Significant alterations were observed in the alanine, aspartate and glutamate metabolism pathway, arginine and proline metabolism pathway, as well as in specific intermediary metabolites such as GABA and taurine. The data thus indicates that BMAA preferentially interferes with fundamental metabolic pathways related to neurotransmission. Importantly, the observed perturbations in amino acid/neurotransmitter metabolism pathways were not related to acute excitotoxicity or oxidative stress but may be of importance for long-term neurodegenerative changes. Notably, changes in amino acid availability can selectively alter the expression of genes and related proteins. Further studies on the effects of BMAA on gene and protein expression are needed to characterize the effects of BMAA in human neuronal cells and in the mammalian brain. Moreover, detailed analysis of the exo-metabolome could potentially provide more information in regards to understanding the complexity of BMAA exposure.
